# Mechanism of weight regain – The role of epigenetic modification in adipocytes

**DOI:** 10.1016/j.apsb.2025.02.002

**Published:** 2025-02-10

**Authors:** Yue Zhao, Xiwen Ma, Jianping Ye

**Affiliations:** a Institute of Trauma and Metabolism, Zhengzhou Central Hospital Affiliated to Zhengzhou University, Zhengzhou 450007, China; b Tianjian Laboratory of Advanced Biomedical Sciences, Academy of Medical Sciences, Zhengzhou University, Zhengzhou 450001, China

**Keywords:** Weight regain, Epigenetic modification, Weight loss, Obesity, Adipose tissue

Weight regain (WR) is a significant challenge in clinical treatment of obesity after successful weight loss. WR happens frequently after weight loss (WL) by dietary or life style adjustment although it also occurs in WL by bariatric surgery and medications^1^, ^2^, ^3^. A general mechanism of WR is “obesity memory”, which refers to a stable gene expression pattern in adipose tissue from obesity. However, the molecular mechanisms underlying obesity memory remain unclear. Recently, Hinte et al.^1^ reported that obesity memory is mediated by an epigenetic mechanism in adipocytes of adipose tissue in a *Nature* article.

Adipose regain is the primary cause of weight rebound. Adipose tissue is the primary organ for energy storage, in which adipocytes take glucose as well as fatty acids, and convert them into triglycerides for energy storage. Additionally, adipose tissue has very important endocrine functions, capable of secreting various hormones in the regulation of energy metabolism, such as leptin and adiponectin^4^. However, excessive fat accumulation in adipocytes leads to adipose tissue expansion under conditions of overweight and obesity. Fat storage in adipocytes is primarily regulated by insulin and sympathetic nerve signals. Insulin promotes the energy storage by induction of glucose and fatty acid uptake through activation of insulin receptor, which then lead to activation of metabolic pathways of *de novo* lipogenesis, triglyceride biosynthesis and lipid droplet formation^2^. These insulin activities promote weight gain through the impact in adipose tissue, which is inhibited by the sympathetic nerve activity through epinephrine (adrenaline), a neurotransmitter and a hormone. Epinephrine activates its receptor in adipocytes to inhibit energy storage through two effects, suppression of lipogenesis and induction of triglyceride breakdown, in favor of energy mobilization and utilization for heat production. This is the physiological basis of sympathetic nerve activity in the induction of insulin resistance under obesity as reported in another recent study^5^. In the physiological conditions, the balance of insulin and sympathetic nerve activities determines weight gain through the actions in adipose tissue. In the condition of energy over intake, disbalance of the two forces leads to adipocyte hypertrophy leading to fat accumulation in WR.

Epigenetic modification represents a new mechanism of obesity memory underlying WR^1^. Current mechanisms for WR include several hypotheses^6^, such as obesity memory in the adipose tissue, energy metabolism adaptation, lipid metabolism disorder, and appetite dysregulation. In the adipose tissue, tissue expansion induces infiltration of inflammatory cells including macrophages, neutrophils, T cells and B cells. Initially, it was believed that increased presence of macrophages in adipose tissue was a key factor in obesity memory, which exhibited an increased secretion of pro-inflammatory cytokines, such as IL-1 and IL-6. These characteristics of immune cells persist in the adipose tissue even after WL^5^, ^6^, ^7^. The immune cells are believed to secrete extracellular matrix-modifying enzymes, and angiogenic factors for adipose tissue remodeling in the tissue expansion, but evidence supporting the possibilities is limited, especially in human WR^6^^,^^8^. The adipose inflammation does not cause systemic insulin resistance in obesity^9^, but rather restricts the tissue expansion through induction of energy expenditure against WR^10^. Therefore, the exact role of adipose inflammation remains to be defined in WR. Metabolic adaptation after WL include a decrease in resting energy expenditure, which may lead to more energy storage in favor of WR^6^. However, there is currently no strong evidence for the metabolic adaptation in WR. WL may decrease fat breakdown and utilization during rest and exercise, causing a reduction in lipid catabolism that exists even after two months of WL, which may contribute to WR^6^. Additionally, appetite dysregulation leading to increased food intake is another factor for weight rebound in the WR patients. Factors affecting appetite include behavioral (such as environmental cues) and biological aspects (hormones, neurons, and metabolism, etc.). The increased ghrelin and decreased levels of leptin and GLP-1 after WL cause an increase in hunger and food reward desires in the brain, thereby increasing energy intake for WR^6^. In the mechanisms mentioned above, gene expression is involved in all cases, while the role of epigenetic modifications has not been established in the control of gene expression. There is no strong *in vivo* evidence proving a direct relationship between epigenetic modifications and WR. The new study addressed the issue by exploring epigenetic modifications in the adipose tissue of human body for WR^1^.

To find new evidence of obesity memory in humans, Hinte et al.^1^ utilized single-cell nuclear transcriptome sequencing (scRNA-seq) to analyze adipose tissue samples (including visceral and subcutaneous) from three groups (healthy weight, obese, obese with surgery-induced WL) of individuals^1^. They found that more than 70% obesity-induced genes were restored to normal level in adipocytes as well as in macrophages after WL. This pattern of change was observed in both visceral fat and subcutaneous fat. However, the down-regulated genes of adipocytes were different from the up-regulated genes in response to WL, with most remaining down-regulated after WL. These decreased genes included *IGF1*, *LPIN1*, *IDH1* and *PDE3A* in the visceral fat, and *IGF1*, *DUSP1*, *GPX3*, and *GLUL* in the subcutaneous fat. Similar patterns of responses were obtained in adipocytes of obese mice after WL. Interestingly, the pathological features (such as insulin sensitivity, hyperinsulinemia, and hyperleptinemia) caused by obesity mostly were improved by WL in the mice. This suggests that WL did not completely restore the gene expression profiles in adipocytes although WL improved systemic insulin sensitivity and glucose metabolism.

Next, they conducted mouse fat scRNA-seq to test the observations in human. Within the epididymal adipose tissue, 15 cell types/populations were identified including adipocytes, adipocyte progenitor cells, immune cells, mesothelial cells, endothelial cells, and epithelial cells, etc. The data showed that adipocytes, adipocyte progenitor cells, endothelial cells, epithelial cells, and macrophages exhibited more pronounced transcriptional dysregulation compared to other cell types in the adipose tissue under obesity. This was mainly characterized by the upregulation of genes associated with lysosomal activity, cellular inflammation, and apoptosis, and the downregulation of genes related to lipid metabolism pathways (such as fatty acid oxidation, synthesis, lipogenesis) in these cell populations. WL restored alterations in most genes in majority of cell types including adipocytes and macrophages. The persisted gene alterations suggest that adipocyte functions were not completely recovered after WL^1^.

Subsequently, the authors explored the potential mechanisms of persistent expression profiles in obesity memory through epigenetic analysis of the mouse epididymal adipocytes using techniques such as CUT&Tag, ATAC-seq, TRAP-seq, and multi-omics factor analysis (MOFA) in combination with transgenic mice. The results revealed that many promoters in the adipocytes of obese mice were repressed relatively to the original active state (H3K4me3 and/or H3K27ac) in the lean mice with an increase in H3K27me3. Histone lysine H3K27 is modified by acetylation and methylation. The acetylation marks activation of gene promoter and the methylation represents suppression of the gene promoter. In adipocytes, some gene promoters repressed by obese remained in inhibition after WL. The genes (such as *Gpam*, *Cyp2e1*, and *Acacb*) were related to adipocyte functions. While, some genes that were activated by obesity remained in active status after WL, those genes are related to inflammation (such as *Icam1*, *Lyz2*, and *Tyrobp*). Through GSEA analysis, the authors confirmed that although the level of inflammation was reduced after WL, the reduction was observed mainly in macrophages, but some inflammatory genes remained active in adipocytes. Overall, these results strongly suggest that after WL, stable epigenetic and transcriptional memory based on histone methylation and acetylation modifications persist in the adipocytes, leading to continuous impairment of adipocyte function. Next, *in vitro* adipocyte experiments revealed that this epigenetic memory affected the nutrient uptake capacity and insulin sensitivity of adipocytes. Compared to the control group, adipocytes in the WL group exhibited enhanced glucose and fatty acid uptake, and adipose-derived stromal vascular cells from this group showed increased insulin sensitivity, accumulating lipids more readily under insulin stimulation over the control group. These findings confirm that stable transcriptional and epigenetic memory exist within adipocytes after WL, which promotes the recovery of adipocyte storage function and drives WR^1^.

Currently, most research on WR are focused on DNA methylation in tissues or whole blood, with less attention to the epigenetic studies of histone proteins in adipocytes. This new study utilizes a group of advanced experimental techniques to investigate the epigenetic mechanisms of obesity memory at the single-cell level, clarifying for the first time that a set of epigenetic modifications of histone proteins was induced in adipocytes in cell type-specific manner by obesity. It reveals that the modifications persisted in adipocytes after WL for sustained gene alteration. The epigenetic modifications contributed to the acceleration of WR through improving insulin sensitivity in adipocytes in favor of energy storage. The findings provide a novel insight into the role of adipocytes in WR. However, the persisted genes were different in adipocytes between human and mice although WR are similar in both models. Therefore, the exact mechanism of obesity memory remains to be explored in the context of epigenetics in human WR.

## Author contributions

Yue Zhao drafted the manuscript. Xiwen Ma and Jianping Ye provided the idea and revised the manuscript.

## Conflicts of interest

The authors declare no conflicts of interest.
